# A latitudinal cline in the Chinook salmon (*Oncorhynchus tshawytscha*) *Clock* gene: evidence for selection on PolyQ length variants

**DOI:** 10.1098/rspb.2008.0524

**Published:** 2008-08-19

**Authors:** Kathleen G O'Malley, Michael A Banks

**Affiliations:** Coastal Oregon Marine Experiment Station, Department of Fisheries and Wildlife, Hatfield Marine Science Center, Oregon State UniversityNewport, OR 97365, USA

**Keywords:** Chinook salmon, *Clock* gene, latitudinal cline, migration timing, PolyQ

## Abstract

A critical seasonal event for anadromous Chinook salmon (*Oncorhynchus tshawytscha*) is the time at which adults migrate from the ocean to breed in freshwater. We investigated whether allelic variation at the circadian rhythm genes, *OtsClock1a* and *OtsClock1b*, underlies genetic control of migration timing among 42 populations in North America. We identified eight length variants of the functionally important polyglutamine repeat motif (PolyQ) of *OtsClock1b* while *OtsClock1a* PolyQ was highly conserved. We found evidence of a latitudinal cline in average allele length and frequency of the two most common *OtsClock1b* alleles. The shorter 335 bp allele increases in frequency with decreasing latitude while the longer 359 bp allele increases in frequency at higher latitudes. Comparison to 13 microsatellite loci showed that 335 and 359 bp deviate significantly from neutral expectations. Furthermore, a hierarchical gene diversity analysis based on *OtsClock1b* PolyQ variation revealed that run timing explains 40.9 per cent of the overall genetic variance among populations. By contrast, an analysis based on 13 microsatellite loci showed that run timing explains only 13.2 per cent of the overall genetic variance. Our findings suggest that length polymorphisms in *OtsClock1b* PolyQ may be maintained by selection and reflect an adaptation to ecological factors correlated with latitude, such as the seasonally changing day length.

## 1. Introduction

Many organisms use day length (photoperiod) as an environmental cue to regulate seasonal changes in behaviour, growth, development, reproduction, dormancy and migration (Pittendrigh 1981; Vaz Nunes & Saunders 1999; Bradshaw & Holzapfel 2001). In plants and animals, the daily molecular oscillator, known as the circadian clock, senses changes in photoperiod and mediates a diverse number of photoperiodic responses such as flowering time in long- and short-day plants (*Arabidopsis*, Kardailsky *et al.* 1999; *Oryza*, Yano *et al.* 2000) as well as hormone secretion in mammals (Siberian hamsters, reviewed in Goldman 2001). Most recently, the circadian rhythm gene, *timeless*, has been shown to affect the incident of diapause, a seasonal event in *Drosophila* (Tauber *et al.* 2007).

A critical seasonal event for anadromous fish such as Chinook salmon (*Oncorhynchus tshawytscha*) is the time at which adults migrate from the ocean to their natal rivers to breed. Intra-annual variability in both water flow and temperature limits access to discrete spawning habitats within these rivers, and consequently, locally adapted migratory populations have evolved low inter-annual variability in their seasonal return time. Thus, the timing of this event is primarily an adaptation to long-term average conditions rather than a proximate response to current conditions. Photoperiod is a stable, long-term environmental cue that fishes could use to coordinate their population-specific migratory runs with these seasonally varying conditions (Quinn & Adams 1996). Previous studies have demonstrated that photoperiod is a key environmental cue for maturation timing in salmonid fish (Beacham & Murray 1988) and because this trait is strongly correlated to migration timing (Quinn *et al.* 2000), it is reasonable to hypothesize that photoperiod is also an important cue for anadromous salmon.

In North America, there is a considerable amount of diversity in the timing of Chinook salmon migration. To facilitate management of these locally adapted populations, fishery biologists categorize individuals as belonging to one of four seasonal runs based on the peak freshwater return time of the population. In a population genetic study of 118 Chinook salmon runs based on neutral markers, Waples *et al.* (2004) found that distinct seasonal runs within a geographical region were genetically more similar to each other than to populations with a similar run time from different geographical regions. These results indicate that run timing has evolved multiple times in this species. In the interior Columbia River Basin, however, Waples *et al.* (2004) and others have concluded that run timing differences reflect a much older divergence of two major lineages.

One notable trend throughout the geographical range of Chinook salmon is an increase in run time diversity with decreasing latitude. For instance, primarily summer run Chinook salmon populations occupy the northern rivers of Alaska and Canada while autumn, spring and summer run populations inhabit regions to the south with multiple run time populations coexisting in the same river. The Sacramento River in California, for example, supports three seasonal runs: autumn, spring and the only remaining winter run population. Thus, there appears to be a latitudinal cline in run time diversity of Chinook salmon populations along the west coast of North America.

We previously characterized two copies of the circadian rhythm gene, *Clock*, (*OtsClock1a* and *OtsClock1b*) as potential candidate loci for migration time in Chinook salmon (O'Malley & Banks 2008). The *Clock* gene, which codes for one of the most essential proteins of the circadian oscillator (Lowrey & Takahashi 2004), has been characterized in several model organisms including mouse (King *et al.* 1997), *Drosophila* (Allada *et al.* 1998) and zebrafish (Whitmore *et al.* 1998). Heterodimerization of CLOCK and a second protein, BMAL, produces a transcription-activating complex that regulates the expression of two additional circadian genes, *Period* and *Cryptochrome* (Reppert & Weaver 2002). A critical domain of CLOCK, which affects the transcription-activating potential of this protein, is the carboxyl-terminal polyglutamine repeat motif (PolyQ; Darlington *et al.* 1998). Expansion or contraction in the number of glutamine repeats in this region directly affects the corresponding gene product and thereby influences the circadian phenotype (Gekakis *et al.* 1998).

In an earlier investigation, we studied seasonal migratory runs of Chinook salmon from two systems and showed that length variation in the *OtsClock1b* PolyQ domain provides evidence for potentially adaptive genetic differentiation (O'Malley *et al.* 2007). Furthermore, tests for selective neutrality revealed that *OtsClock1b* deviated from neutral expectations in both systems indicating that this candidate gene is likely under selection. Thus, these results suggest that *OtsClock1b* may influence migration timing of Chinook salmon in these two systems.

Here we screened for length polymorphisms in *OtsClock1a* and *OtsClock1b* PolyQ domains among 42 runs of Chinook salmon and investigated whether there was any association between variation in allele frequency and migration time across a broad latitudinal gradient along the west coast of North America. We then compared these results to data from microsatellite loci presumed to be selectively neutral.

## 2. Material and methods

### (a) Populations of Chinook salmon

**Figure 1:**
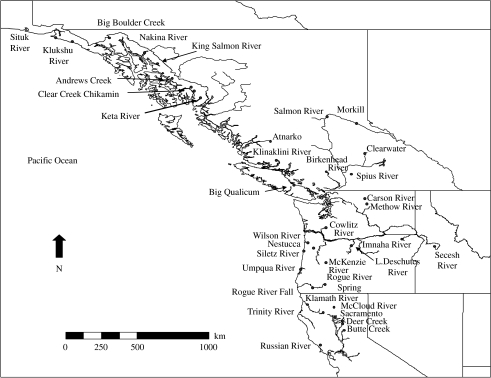
Map showing the location of the 42 Chinook salmon populations from California to Alaska.

Of the 42 populations included in this study, 40 are part of a standardized DNA collection established by the Chinook Technical Committee (CTC) of the Pacific Salmon Commission (figure 1; Seeb *et al.* 2007). Individuals from all CTC populations were previously genotyped at 13 microsatellite loci. For this study, we obtained DNA samples of approximately 48 individuals from each of the 40 populations. In June 2004, we collected 20 Chinook salmon liver samples from the remaining two populations, Alaska's Montana Creek and Situk River, and extracted genomic DNA using the DNeasy Tissue Kit (Qiagen).

### (b) Genotyping of OtsClock1a and OtsClock1b PolyQ domains

We designed oligonucleotide primers to amplify a 199 bp fragment encompassing the polyglutamine repeat motif of *OtsClock1a*. The fluorescently labelled sense primer was 5′-GGTTCCTAATGTAGTTCCTGTGCTT-3′ and 5′-GATTTCTCACCTGGACACTGGGCT-3′ the antisense. We used previously designed oligonucleotide primers to amplify a 335 bp fragment of the *OtsClock1b* PolyQ domain gene (O'Malley *et al.* 2007).

DNA was amplified in 5 μl reactions using two touchdown PCR profiles: one initial denaturing cycle of 3 min at 94°C, followed by one cycle of 1 min at 94°C, 1 min at 62°C annealing temperature, and 1 min 30 s at 72°C. In subsequent cycles, the annealing temperature was decreased by 2°C until 52°C was reached for *OtsClock1a* and 56°C for *OtsClock1b*, followed by 29 more cycles of 1 min at 94°C, 1 min at 52°C/56°C, 1 min 30 s at 72°C, and a final extension of 10 min at 72°C. PCR products were electrophoresed on an Applied Biosystems 3730XL DNA Analyzer and scored as length polymorphisms using GeneMapper software.

### (c) Sequencing of OtsClock1a and OtsClock1b PolyQ domain

To determine nucleotide sequence of the *Clock* PolyQ length variants, we PCR amplified the region from individuals of known genotype (table 1). PCR products were excised from 1.5 per cent agarose gels and purified using QIAquick gel extraction kit (Qiagen). Purified PCR products were then cloned into pCR4-TOPO vector using the TOPO TA Cloning kit for Sequencing (Invitrogen). Plasmid DNA was isolated using Wizard Plus SV Minipreps (Promega) and sequenced using Big Dye Terminator v. 3.1 Cycle Sequencing Ready Reaction. All sequences were generated on an Applied Biosystems 3730XL DNA Analyzer. Multiple sequence alignments were created manually using BioEdit Sequence Alignment Editor (Hall 1997) and automatically using ClustalW (Thompson *et al.* 1994).

### (d) Data analysis

Calculations of allelic frequencies were performed using Genepop v. 3.3 (Raymond & Rousset 1995). We calculated pairwise estimates of *F*_st_ and permuted the data 1000 times using Genetix v. 4.02 (Belkhir 2000). Associations between latitude and allele frequency as well as average allele length were examined using linear regression (SigmaPlot v. 6.0). Average allele length is defined as the sum of all allele lengths of individuals in a given population divided by the total number of individuals in that population. To determine if regression coefficients for the candidate gene alleles differed significantly from neutral expectations, we compared these values to a distribution of regression coefficients (allele frequency on latitude) for 441 alleles (13 microsatellite loci) from the CTC microsatellite baseline dataset.

Partial Mantel tests of association were also used to analyse the patterns of spatial variation by comparing matrices of genetic distance (i.e. *F*_st_, allele frequency, average allele length) to geographical distance (i.e. latitude, longitude). Mantel tests were performed with 10 000 permutations using the software program Passage (Rosenberg 2001).

To partition the overall *F*_st_ estimate into genetic variance related to run timing, geographical region and differences among populations, we performed two hierarchical gene diversity analyses (Lewis & Zaykin 2001). The first analysis was based on allelic variation at candidate genes while the second was based on variation at 13 presumed neutral microsatellite loci. To perform the hierarchical analysis, we grouped the 40 populations into geographical regions within one of three run times (table 2). Run time is defined as the peak return time of a given population to freshwater spawning grounds. We primarily used run time designations listed in Waples *et al.* (2004) and Seeb *et al.* (2007). To obtain a balanced dataset, the McCloud River winter and Big Qualicum autumn runs were excluded from each analysis. In addition, three populations were excluded from the microsatellite analysis owing to lack of data (Wilson Fall, Siletz Fall and Montana summer).

## 3. Results

### (a) Conservation of the OtsClock1a PolyQ domain

**Figure 2:**
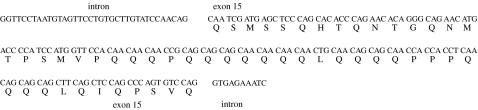
Nucleic and amino acid sequence of *OtsClock1a* PolyQ locus.

We found no evidence of length polymorphism in the *OtsClock1a* PolyQ domain among the Chinook salmon populations as all individuals were homozygous for the 199 bp allele. The *OtsClock1a* PolyQ fragment consists of 52 amino acid residues flanked by 36 and 11 bp of non-coding sequence (figure 2).

### (b) Length variation of the OtsClock1b PolyQ domain

We identified eight length variants of the *OtsClock1b* PolyQ domain among 42 Chinook salmon populations ranging from California to Alaska. Two length variants, 335 and 359 bp, are most common with frequencies of 0.737 and 0.225, respectively. Two less common variants, 338 and 383 bp, have frequencies of 0.010 and 0.026, respectively. The combined frequency of the remaining four alleles, 293, 329, 356, and 362 bp, is 0.002 (table 3). We identified 16 genotypic classes among the 42 populations. The frequency of the four most common genotypes are: 335/335 (0.56); 335/359 (0.29); 359/359 (0.07); and 335/383 (0.03). The remaining 12 genotypic classes (293/335, 329/329, 329/335, 329/335, 329/359, 335/338, 338/338, 338/359, 335/356, 359/362, 359/383 and 383/383 bp) have a frequency less than or equal to 0.01.

**Figure 3:**
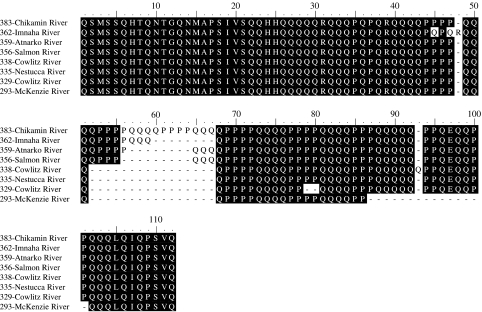
Multiple sequence alignment of the eight *OtsClock1b* length variants. Allele length (base pairs) and source population is listed for each sequence.

Length variation of the eight *OtsClock1b* PolyQ alleles (80–110aa) is primarily characterized by the insertion and deletions consisting of both glutamine (Q) and proline (P) repeats (figure 3). Two exceptions include allele 293 bp, which lacks one glutamic acid and allele 362 bp, which contains one additional arginine residue and two unique and non-conservative amino acid changes (P–Q).

### (c) Latitudinal distribution of OtsClock1b PolyQ alleles compared to microsatellites

**Figure 4:**
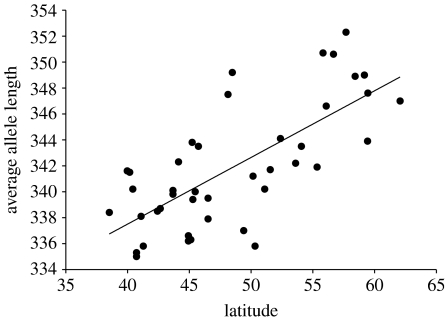
Plot of latitude in degrees (*x*-axis) against average allele length. (*R*^2^=0.516, *p*=0.0001; *y*=316.9+0.51*x*).

**Figure 5:**
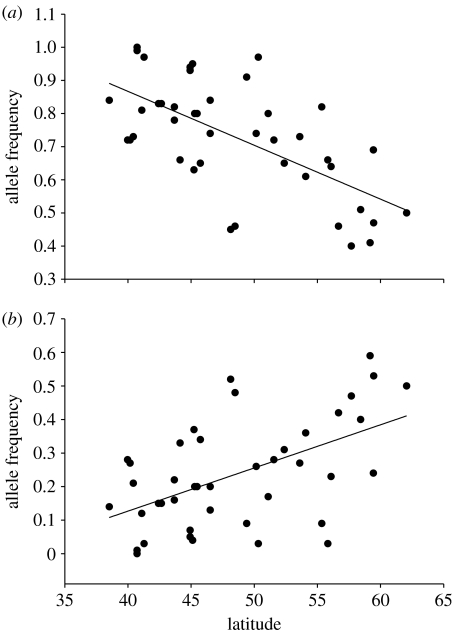
Plot of latitude in degrees (*x*-axis) against frequencies of alleles for (*a*) 335 bp (*R*^2^=0.407, *p*=0.0001; *y*=1.52+−0.016*x*) and (*b*) 359 bp (*y*-axis) (*R*^2^=0.285, *p*=0.0003; *y*=−0.39+0.012*x*).

We found a significant association between average allele length of *OtsClock1b* PolyQ variants and latitude using linear regression (*R*^2^=0.516, *p*<0.0001, regression coefficient=0.514; figure 4). Examination of the two most common *OtsClock1b* alleles (335 and 359 bp) revealed a significant association between frequency and latitude albeit in the opposite direction. The short 335 bp allele increases in frequency at lower latitudes (*R*^2^=0.407, *p*<0.0001, regression coefficient=−0.016) while the long 359 bp allele increases in frequency at higher latitudes (*R*^2^=0.285, *p*=0.0003, regression coefficient=0.013) (figure 5*a*,*b*).

To test for departure from neutral expectations, we calculated the regression coefficient of allele frequency on latitude for each of the 441 microsatellite alleles and compared these values to those for the two candidate alleles. The regression coefficients for 335 bp (−0.016) and 359 bp (0.012) were exceeded by less than 1 per cent of the microsatellites.

To examine the spatial distribution of *OtsClock1b* alleles further, we performed partial Mantel tests of association and compared allele frequency (335 and 359 bp), average allele length and pairwise *F*_st_ estimates to latitude and longitude. We found a significant positive association between all three measures of genetic distance (allele frequency, average length and *F*_st_ estimates) and latitude (constant=longitude; table 4). By contrast, we found no significant association between the three genetic distance measures and longitude (constant=latitude) indicating that the major directional component of the observed clinal patterns is north–south (table 4).

We discovered an analogous pattern of geographical distribution for the two less common length variants (338 and 383 bp). The short 338 bp allele is only present in 12 populations located south of 48° latitude while the long 383 bp allele is present in 9 populations located north of 48° latitude. One exception to this distinct geographical separation occurs in the Umpqua River, where two individuals have a 383 bp allele. In summary, the overall trend in *OtsClock1b* allelic variation among North American Chinook salmon populations is the prevalence of long *OtsClock1b* PolyQ alleles in northern populations (359 and 383 bp) and short PolyQ alleles (335 and 338 bp) in southern populations.

### (d) Genetic structure based on OtsClock1b PolyQ alleles compared to microsatellites

The overall *F*_st_ estimate among the 42 populations based on *OtsClock1b* was 0.142 while the overall *F*_st_ estimate based on microsatellite loci was 0.073. We found no significant association between pairwise *F*_st_ estimates based on *OtsClock1b* allele frequency and pairwise *F*_st_ estimates based on microsatellite allele frequencies (Mantel test: *r*=0.037, *p*=0.409).

A hierarchical gene diversity analysis of *OtsClock1b* PolyQ variation showed that genetic variance related to run timing was 0.056, indicating that run timing explained 40.9 per cent of the overall genetic differences among populations. Genetic variance related to differences among populations was 0.065 (47.4%) while the remaining 0.016 (11.7%) resulted from differences among regions. By contrast, a hierarchical gene diversity analysis of 13 microsatellite loci revealed that genetic variance related to run timing was 0.010, indicating that run timing only explained 13.2 per cent of the overall genetic differences among populations. Genetic variance related to differences among populations was 0.057 (75.0%) while the remaining 0.0096 (11.8%) resulted from differences among regions.

## 4. Discussion

### (a) Characterization of duplicated Clock PolyQ domains

The PolyQ region corresponds to the transactivation domain of the CLOCK protein. Studies have demonstrated that length variation in this glutamine-rich region can affect the binding affinity of this transcription factor and thereby alter the circadian phenotype (Darlington *et al.* 1998). This study is one of a limited number investigating repeat length expansion of the *Clock* PolyQ domain in natural populations (birds: Johnson *et al.* 2007; *Drosophila*: Saleem *et al.* 2001, Weeks *et al.* 2006; naked mole rat: Avivi *et al.* 2001). We identified eight length variants of *OtsClock1b* PolyQ while the *OtsClock1a* domain was highly conserved. We previously discovered that a 1200 bp fragment located downstream of the *OtsClock1a* PolyQ shows a 91 per cent sequence identity to the Atlantic salmon *Transferrin* gene (O'Malley & Banks 2008). Considering that this fragment is not present in *OtsClock1b*, it is possible that strong purifying selection may inhibit repeated expansion of the *OtsClock1a* PolyQ domain.

### (b) Latitudinal cline of OtsClock1b maintained by selection

DeBruyne *et al.* (2006) discovered that *Clock* plays a central role in the light input pathway of the circadian timing mechanism. We found evidence for a latitudinal cline in average allele length as well as frequency of the two most common alleles; the longer variant increasing in frequency in northern latitudes while the shorter variant increases in frequency in southern latitudes. Comparing the frequency distribution of these two *OtsClock1b* PolyQ alleles with presumably selectively neutral microsatellite alleles strongly suggests that this latitudinal cline is maintained by selection. We also found an analogous geographical pattern in the frequency distribution of the two less common alleles with the longer variant present primarily in northern populations (greater than 48°) while the shorter variant was only present in southern populations (less than 48°). We hypothesize that the observed clinal variation in *OtsClock1b* PolyQ alleles could reflect an adaptation to photoperiodic parameters correlated with latitude as the seasonal variation in day length is more pronounced in Alaska compared to California. A recent study by Johnson *et al.* (2007) detected a similar latitudinal cline in the avian *Clock* PolyQ domain (longer alleles in north, shorter alleles in south), which the authors propose may be indicative of local adaptation to latitudinal gradients in the seasonal rate of change of photoperiod.

Pittendrigh *et al.* (1991) first observed that the amplitude of the circadian pacemaker declines as the duration of the entraining light pulse (photoperiod) is increased, ultimately producing a latitudinal cline in nature. More recently, Vitaterna *et al.* (2006) demonstrated that a mutation in the *Clock* PolyQ domain modifies the amplitude of the pacemaker which, in turn, can have significant effects on the entrainment behaviour of organisms to light and other resetting stimuli. For Chinook salmon, the duration of the daily photoperiod throughout the breeding season is steadily increased as one moves north. Natural length variation in the *OtsClock1b* PolyQ may also modify the circadian pacemaker amplitude such that selection for the conservation of pacemaker amplitude during the breeding season may produce the observed latitudinal cline in *OtsClock1b* allele frequency.

Other ecological factors correlated with latitude may also contribute to the clinal variation in *OtsClock1b* PolyQ alleles as has been demonstrated for the threonine–glycine-encoding (Thr–Gly) repeat region of the *Drosophila* circadian rhythm gene, *PERIOD* (Costa *et al.* 1991; Castiglione-Morelli *et al.* 1995; Sawyer *et al.* 1997; Rosato & Kyriacou 2001; Sawyer *et al.* 2006). Similar to our discoveries for *OtsClock1b*, two alleles make up approximately 90 per cent of the natural variation in the *Drosophila PERIOD* gene, (Thr–Gly)_20_ and (Thr–Gly)_17_, with the longer allelic variant predominating in the northern Europe and the shorter variant predominating in the southern region (Costa *et al.* 1992). This clinal variation of the *Drosophila Period* gene is thought to be maintained by climate-related selection, as temperature compensation of the circadian clock differs among the Thr–Gly variants (Sawyer *et al.* 1997). (Thr–Gly)_17_ variants show a 24 hour cycle at higher temperatures but at a shorter period as temperatures are decreased. By contrast, the (Thr–Gly)_20_ variants are not sensitive to changes in temperature and show, on average, a period slightly shorter than 24 hours. Therefore, the two major (Thr–Gly) variants appear to be adapted to the thermal environments in which they predominate, (Thr–Gly)_17_ in southern and (Thr–Gly)_20_ in northern Europe.

Temperature is a critical environmental variable particularly during the freshwater stages of Chinook salmon life history. Seasonal fluctuations in temperature can create thermal barriers to salmon migrating to freshwater spawning habitats (Richter & Kolmes 2005). Consequently, populations have adapted to long-term average conditions for specific rivers throughout their geographical range. It is plausible that climate-related selection may contribute to the latitudinal cline in *OtsClock1b* PolyQ variants as has been documented for the *Drosophila Period* gene. However, the relationship between the *Clock* gene and temperature has yet to be explored in any model organism.

The primary aim of this study was to investigate whether there was any association between variation in allele frequency and migration timing among Chinook salmon populations along the west coast of North America. Considering the clinal variation in run time diversity, one would predict increasing gene diversity in the southern populations. We, however, found no evidence for this trend. In fact, California's Trinity autumn and Trinity spring populations were essentially fixed for the 335 bp allele (frequency=1.00 and 0.99, respectively).

### (c) Contrasting population genetics based on OtsClock1b compared to microsatellites

Pairwise *F*_st_ values based on *OtsClock1b* were not significantly associated with those based on microsatellites indicating that they do not reflect similar patterns of population structure and/or history. Thus, while we cannot rule out the possibility that the spatial distribution of *OtsClock1b* allelic variants is influenced by historical separations, our findings provide evidence for clinal variation probably maintained by selection.

The hierarchical gene diversity analysis of eight *OtsClock1b* PolyQ variants revealed that almost half of the overall genetic variance is related to run timing (40.9%) with the remaining variation attributed to differences among regions and populations (11.7 and 47.4%, respectively). These results contrast sharply with those obtained for assumed neutral microsatellite loci, which indicate that run timing explains only 13.2 per cent of the overall genetic variance. Our microsatellite findings are in accord with those reported by Waples *et al.* (2004) where only 10.2 per cent of the overall genetic variance among 118 Chinook salmon populations was explained by run timing based on allozymes.

We discovered several examples where temporally divergent runs within a river have similar *OtsClock1b* allele frequencies (i.e. Trinity, Rogue, Umpqua and Siletz Rivers). Therefore, the large percentage of genetic variance attributed to run timing is likely a reflection of the strong latitudinal cline in allele frequency as primarily summer runs inhabit the northern range of this species where average allele length is large. To test this, we excluded the summer run populations and reanalysed data from 20 autumn and spring run populations in Oregon, Washington and California and found that the overall genetic variance related to run timing decreased to 20 per cent while the differences among populations increased to 79 per cent. Thus, other environmental factors or life-history traits correlated with latitude probably confound the analysis.

### (d) Summary

Duplicated *Clock* genes in Chinook salmon show distinct patterns of length variation in the functionally significant PolyQ domain. In contrast to the highly conserved PolyQ of *OtsClock1a*, we identified eight length variants of the *OtsClock1b* PolyQ domain. Based on the *OtsClock1b* PolyQ variation, we found evidence for a latitudinal cline in average allele length and frequency as well as a large component of genetic variance explained by run timing. These results contrast sharply with those obtained for presumed selectively neutral microsatellite markers suggesting that the observed variation may be maintained by selection and reflect an adaptation to ecological factors correlated with latitude such as photoperiod.

## Figures and Tables

**Table 1:** Allele, population, geographical location and run time information of the individuals used to determine nucleotide sequence of the eight novel alleles are listed.

allele	population	latitude	longitude	run time
293	McKenzie River, OR	44.12	122.62	S
329	Cowlitz River, WA	46.51	122.62	F
335	Salmon River, BC	54.07	122.55	S
	Cowlitz River, WA	46.51	122.62	F
	McKenzie River, OR	44.12	122.62	S
338	Cowlitz River, WA	46.51	122.62	F
356	Salmon River, BC	54.07	122.55	S
359	Atnarko River, BC	52.37	126.10	Su
362	Imnaha River, OR	45.73	117.86	S
383	Chikamin, AK	55.82	130.89	Su
	Atnarko River, BC	52.37	126.10	Su
	S. Umpqua River, OR	43.67	124.20	F

**Table 2:** Hierarchy used to partition the overall *F*_st_ into genetic variance related to run timing, region and differences among rivers.

*autumn run*					
					
California	Oregon	Columbia River			
Battle Creek	Umpqua River	Cowlitz River			
Russian River	Siletz River	L. Deschutes River			
Klamath River	Nestucca River				
Trinity River	Wilson River				
	Rogue River				

*spring run*					
					
California	Oregon	Columbia River	British Columbia		
Butte Creek	Umpqua River	Cowlitz River	Spius River		
Deer Creek	Siletz River	Carson River	Birkenhead River		
Trinity River	Rogue River	McKenzie River	Salmon River		

*summer run*					
					
Columbia/Snake Rivers	S SE Alaska	SE Alaska	N SE Alaska	Interior BC	Coastal BC
Methow River	Chikamin River	Andrews Creek	Big Boulder Creek	Clearwater	Klinaklini River
Imnaha River	Clear Creek	King Salmon River	Klukshu River	Morkill	Atnarko
Secesh River	Keta River	Nakina River	Situk River		
			Montana Creek		

**Table 3:** OtsClock1b PolyQ variant frequencies in 42 populations of Chinook salmon (*O. tshawytscha*). Latitude/longitude (degrees), number of alleles (*N*),and run time (autumn, A; winter, W; spring, S; summer, Su) are listed for each population.

population	run time	°latitude	°longitude	*N*	293	329	335	338	356	359	362	383
Montana River, AK	Su	62.06	150.04	40	0.00	0.00	0.50	0.00	0.00	0.50	0.00	0.00
Situk River, AK	Su	59.45	139.57	40	0.00	0.00	0.47	0.00	0.00	0.53	0.00	0.00
Big Boulder Creek, AK	Su	59.43	136.19	86	0.00	0.00	0.69	0.00	0.00	0.24	0.00	0.07
Klukshu River, AK	Su	59.17	138.53	82	0.00	0.00	0.41	0.00	0.00	0.59	0.00	0.00
Nakina River, AK	Su	58.42	133.99	88	0.00	0.00	0.51	0.00	0.00	0.40	0.00	0.09
King Salmon River, AK	Su	57.68	133.17	96	0.00	0.00	0.40	0.00	0.00	0.47	0.00	0.13
Andrews Creek, AK	Su	56.67	132.25	86	0.00	0.00	0.46	0.00	0.00	0.42	0.00	0.12
Clear Creek, AK	Su	56.08	131.07	64	0.00	0.00	0.64	0.00	0.00	0.23	0.00	0.13
Chikamin, AK	Su	55.82	130.89	90	0.00	0.00	0.66	0.00	0.00	0.03	0.00	0.31
Keta River, AK	Su	55.34	130.48	80	0.00	0.00	0.82	0.00	0.00	0.09	0.00	0.09
Salmon River, BC	S	54.07	122.55	88	0.00	0.02	0.61	0.00	0.01	0.36	0.00	0.00
Morkill, BC	Su	53.60	120.70	90	0.00	0.00	0.73	0.00	0.00	0.27	0.00	0.00
Atnarko, BC	Su	52.37	126.10	84	0.00	0.00	0.65	0.00	0.00	0.31	0.00	0.04
Clearwater, BC	Su	51.55	120.20	86	0.00	0.00	0.72	0.00	0.00	0.28	0.00	0.00
Klinaklini River, BC	Su	51.10	125.72	80	0.00	0.00	0.80	0.00	0.00	0.17	0.00	0.03
Birkenhead River, BC	S	50.32	122.60	94	0.00	0.00	0.97	0.00	0.00	0.03	0.00	0.00
Spius River, BC	S	50.15	121.02	50	0.00	0.00	0.74	0.00	0.00	0.26	0.00	0.00
Big Qualicum, BC	F	49.40	124.62	94	0.00	0.00	0.91	0.00	0.00	0.09	0.00	0.00
Carson River, WA	S	48.48	120.19	54	0.00	0.00	0.46	0.00	0.00	0.48	0.00	0.06
Methow River, WA	Su	48.13	120.06	58	0.00	0.00	0.45	0.03	0.00	0.52	0.00	0.00
Cowlitz River, WA	A	46.51	122.62	86	0.00	0.02	0.84	0.01	0.00	0.13	0.00	0.00
Cowlitz River, WA	S	46.51	122.62	70	0.00	0.06	0.74	0.00	0.00	0.20	0.00	0.00
Imnaha River, OR	S	45.73	117.86	82	0.00	0.00	0.65	0.00	0.00	0.34	0.01	0.00
Wilson River, OR	A	45.47	123.75	72	0.00	0.00	0.80	0.00	0.00	0.20	0.00	0.00
L. Deschutes River, OR	A	45.28	121.02	76	0.00	0.00	0.80	0.00	0.00	0.20	0.00	0.00
Secesh River, ID	S	45.23	115.81	76	0.00	0.00	0.63	0.00	0.00	0.37	0.00	0.00
Nestucca River, OR	A	45.12	123.40	92	0.00	0.00	0.95	0.01	0.00	0.04	0.00	0.00
Siletz River, OR	S	44.92	124.02	64	0.00	0.00	0.94	0.02	0.00	0.05	0.00	0.00
Siletz River, OR	A	44.92	124.02	60	0.00	0.00	0.93	0.00	0.00	0.07	0.00	0.00
McKenzie River, OR	S	44.12	122.62	86	0.01	0.00	0.66	0.00	0.00	0.33	0.00	0.00
S. Umpqua River, OR	A	43.67	124.20	94	0.00	0.00	0.82	0.00	0.00	0.16	0.00	0.02
N. Umpqua River, OR	S	43.67	124.20	88	0.00	0.00	0.78	0.00	0.00	0.22	0.00	0.00
Rogue River, OR	S	42.65	122.68	46	0.00	0.00	0.83	0.02	0.00	0.15	0.00	0.00
Rogue River, OR	A	42.42	123.45	48	0.00	0.00	0.83	0.02	0.00	0.15	0.00	0.00
Klamath River, CA	A	41.27	123.78	90	0.00	0.00	0.97	0.00	0.00	0.03	0.00	0.00
McCloud River, CA	W	41.09	122.12	188	0.00	0.00	0.81	0.07	0.00	0.12	0.00	0.00
Trinity River, CA	A	40.72	122.80	94	0.00	0.00	1.00	0.00	0.00	0.00	0.00	0.00
Trinity River, CA	S	40.72	122.80	82	0.00	0.00	0.99	0.00	0.00	0.01	0.00	0.00
Sacramento, CA	A	40.42	122.03	182	0.00	0.00	0.73	0.06	0.00	0.21	0.00	0.00
Deer Creek, CA	S	40.17	121.60	156	0.00	0.00	0.72	0.01	0.00	0.27	0.00	0.00
Butte Creek, CA	S	39.97	121.58	36	0.00	0.00	0.72	0.00	0.00	0.28	0.00	0.00
Russian River, CA	A	38.52	122.98	64	0.00	0.00	0.84	0.02	0.00	0.14	0.00	0.00
total (*N*)	—	—	—	3462	0.0002(1)	0.002(8)	0.737(2550)	0.010(33)	0.0002(1)	0.225(779)	0.0002(1)	0.026(89)

**Table 4:** Partial Mantel tests of association between *OtsClock1B* PolyQ genetic distance and geographical distance. (*p*<0.05 are shown in italic.)

genetic matrices	distance matrices			
			
		constant	*r*	*p*
*F*_st_	latitude	longitude	0.2397	*0.0005*
	longitude	latitude	0.0478	0.6315
allele frequency 335	latitude	longitude	0.2879	*0.0003*
	longitude	latitude	0.0188	0.8569
allele frequency 359	latitude	longitude	0.1783	*0.0037*
	longitude	latitude	0.1492	0.0672
average allele length	latitude	longitude	0.4032	*0.0001*
	longitude	latitude	−0.0989	0.2434
